# Technological approach to invasive species: the role of remote sensing and predictive modelling

**DOI:** 10.1007/s10661-026-16070-x

**Published:** 2026-10-31

**Authors:** Ayushi Gupta, Damanti Murmu, Prakash Rajak, Leesh Ray, Akhilesh Singh Raghubanshi, Hema Singh, Abhinav Wadhwa

**Affiliations:** 1 Ecosystems Analysis Laboratory, Department of Botany, Institute of Science, Banaras Hindu University, Varanasi, India; 2 Institute of Environmental Science and Development, Banaras Hindu University, Varanasi, India; 3 Ashoka Trust for Research in Ecology and the Environment (ATREE) Bengaluru, Bengaluru, Karnataka India; 4 Discovery Partners Institute, The Grainger College of Engineering, University of Illinois Urbana Champaign, Urbana Champaign, IL USA

**Keywords:** Invasive plant species, Remote sensing, Predictive modelling, Hyperspectral imagery, Machine learning, Uncrewed aerial systems, Biodiversity, Sustainable Development Goals

## Abstract

Remote sensing, predictive modelling and machine learning are increasingly central to detecting and monitoring invasive plant species across broad and heterogeneous landscapes. This review synthesises 25 years of remote sensing–based invasive plant species research (2000–2025) to determine how sensor selection, analytical methods and reporting practices have evolved. Records from Web of Science and Scopus were screened using defined eligibility criteria and coded by platform, sensor, species, region, method, impact category, accuracy reporting and linkage with the United Nations Sustainable Development Goals. Bibliometric keyword analysis was used to examine thematic structure and emerging research directions. The evidence shows rapid publication growth after 2015, continued dominance of satellite data, increasing use of uncrewed aerial systems, light detection and ranging, synthetic aperture radar and machine learning classifiers and persistent geographic imbalance. Of 166 retained studies, 149 provided extractable impact information and 76 reported overall accuracy values. The literature indicates a shift from descriptive mapping toward integrated monitoring, risk assessment and decision support frameworks for biodiversity conservation, restoration planning and invasive plant management.

**Graphical Abstract:**

**Figure:**
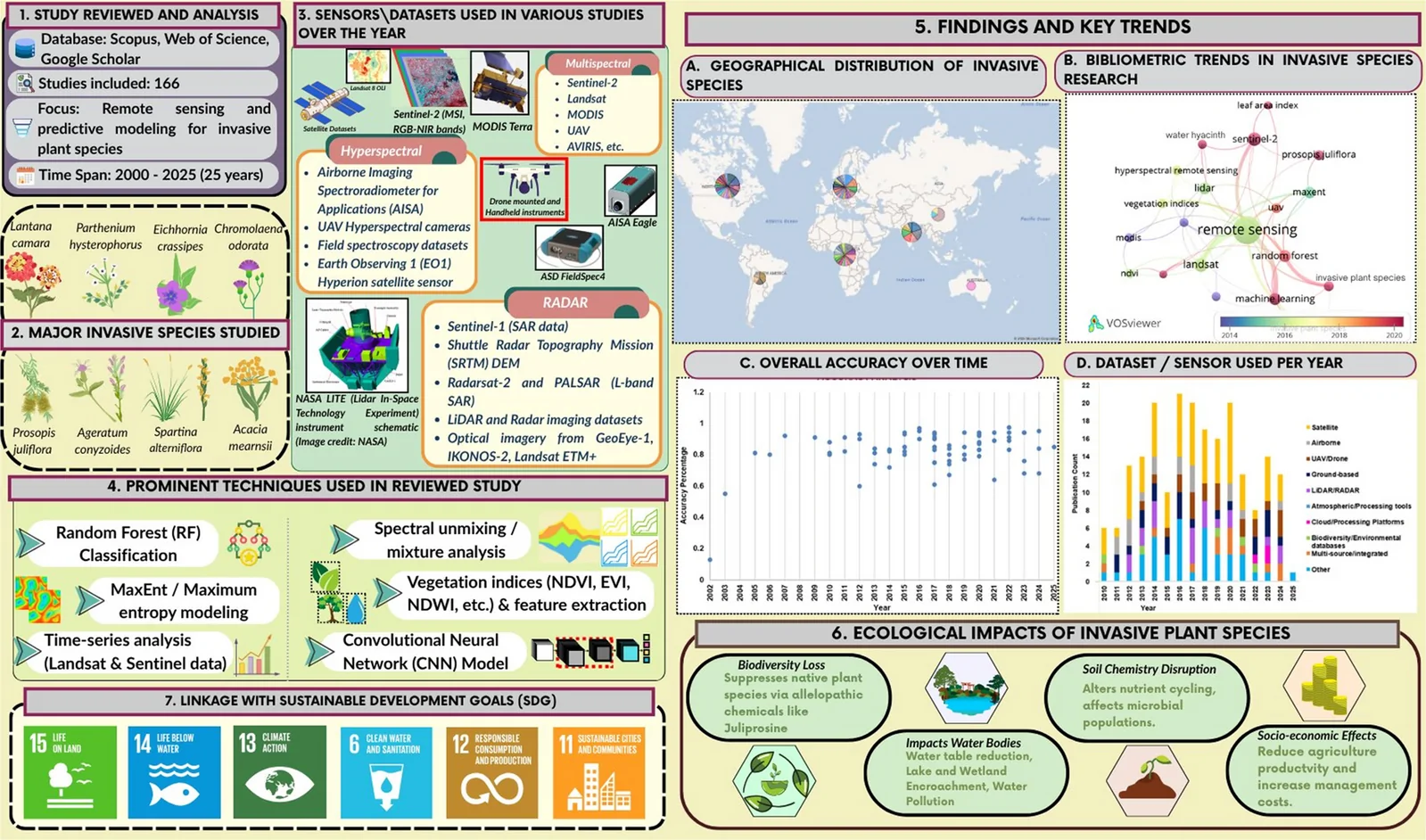

**Supplementary Information:**

The online version contains supplementary material available at https://doi.org/10.1007/s10661-026-16070-x.

## Introduction

Globally, biological invasions pose a serious and increasing threat to ecosystem function, biodiversity, human welfare and economic stability. Habitat degradation and biodiversity loss can result when invasive plant species (IPS) substantially alter ecosystem structure and function (Simberloff & Rejmánek, 2011). According to Qian and Ricklefs (2006), IPS can reduce genetic, species and ecosystem diversity by disrupting biotic interactions and homogenising ecological communities across large spatial scales. Such invasions have substantial economic and ecological effects, with annual global costs estimated at USD 423 billion (Diagne et al., 2021). Human activities such as trade, aquaculture and urbanisation are major contributors to the spread of IPS worldwide (Blanchard et al., 2023). These frequently inadvertent introductions enable aggressive species to proliferate and outcompete native plants for essential resources. Invasive plant species can alter soil composition, interfere with hydrological cycles, degrade habitats and weaken ecosystem resilience (Vilà et al., 2011). By competing for light, space and nutrients, IPS also threaten endemic species, including mangrove trees in coastal ecosystems (Arasumani et al., 2025; Biswas et al., 2018; Zhou et al., 2015). These effects highlight the need for early detection and rapid response to minimise biodiversity loss and maintain ecosystem integrity (Bellard et al., 2016; Delgado et al., 2016; Seebens et al., 2017).

The effects of biological invasions on society and the economy are significant, with IPS such as *Prosopis juliflora*, *Lantana camara* and *Parthenium hysterophorus* disrupting ecological succession, agricultural productivity and water availability (Shackleton et al., 2017). Species like *Eichhornia crassipes*, *Acacia longifolia, Spartina alterniflora*, *Chromolaena odorata* and *Tamarix* spp. are among those listed as problematic by the IUCN Invasive Species Specialist Group (ISSG) Global Invasive Species Database (GISD) database (https://www.iucngisd.org/gisd/), which provides global information on invasive species ecology, distribution, impacts and management. Notably, *Prosopis juliflora* is ranked as one of the 100 worst IPS in the world by the Invasive Species Specialist Group (ISSG), highlighting its potential for substantial ecological impacts (Pagad et al., 2015). While *P. juliflora* does have some beneficial uses, such as providing charcoal, fodder and food, its negative impacts generally outweigh these benefits. Its spread has resulted in declines in native species (Rajak et al., 2025). Remote sensing offers advanced tools to detect, map and track invasive plant species like *P. juliflora*, enabling timely management and mitigation strategies at both local and global scales.

Monitoring the spread of invasive plant species across large areas presents substantial logistical challenges. Traditional field-based mapping is time-consuming, labour-intensive and often impracticable in difficult terrain (Flores-De-santiago & Flores-Verdugo, 2024). Remote sensing (RS) technologies provide spatially explicit observations with repeated coverage over large areas. Spaceborne platforms support efficient monitoring of invasive plant distributions and dynamics (Valderrama-Landeros et al., 2024). Early studies relied mainly on spectral reflectance differences, whereas more recent work incorporates texture and phenology-based detection (Gupta et al., 2022a, 2022b, 2024). Dense time series of vegetation indices can capture seasonal patterns that distinguish invasive from native vegetation (Valderrama-Landeros et al., 2021). In Ethiopia, remote sensing analyses also documented substantial losses in ecosystem services associated with *Prosopis* invasion (Shiferaw et al., 2019).

Recent studies in South African fynbos demonstrate the value of cost-effective uncrewed aerial systems (UAS), machine learning and structure-from-motion products for fine-scale vegetation and biodiversity monitoring. Bhatta et al. (2025) quantified structural differences in low-stature Mediterranean-type shrubland, while Chaity et al. (2026) used high-resolution multispectral imagery and machine learning to classify post-fire vegetation and estimate plant diversity. These studies provide relevant methodological examples from an invasion-prone and underrepresented biome.

Beyond agriculture and biodiversity, invasive plant species may affect public health indirectly by altering habitats used by disease vectors. Remotely sensed vegetation condition, land surface temperature, moisture and habitat structure have supported modelling of malaria-vector density in West Africa (Dambach et al., 2012), tick habitat suitability in north-western Italy (Viani et al. 2024) and Rift Valley fever risk in Africa (Anyamba et al., 2012). These applications demonstrate the wider relevance of remotely sensed environmental information for assessing ecological disturbance and associated health risks.

Time-sensitive RS data can identify many of these species due to their distinctive phenological characteristics (Ouyang et al. 2013; Shen et al., 2021). For instance, *Lantana camara* was distinguished from the dry forest canopy in the Indian subcontinent using dry-season imagery (Prasad, 2008) *Lonicera maackii* was successfully identified using late-season imagery when its green leaves stood out against the senesced native foliage. For efficient mapping and monitoring, the aforementioned examples highlight the value of integrating ecological insights with spectral and temporal RS data. When RS technology is combined with ecological niche modelling, it provides spatially precise invasion risk projections based on environmental suitability (Herborg et al., 2007; Thuiller et al., 2005). Research on invasive plants in temperate, tropical and dry regions increased cinsiderably as a result of the expanding availability of geospatial data, open-access platforms like Google Earth Engine (https://earthengine.google.com/) and large archives like Landsat (https://www.usgs.gov/landsat-missions) and Sentinel (https://sentinels.copernicus.eu/) (Dawson et al., 2013; Essl et al., 2015). Open-access databases and interoperable biodiversity platforms have further strengthened predictive modelling and ecological monitoring frameworks. Databases such as the Global Biodiversity Information Facility (GBIF; https://www.gbif.org/) provide global species occurrence records, while the National Centre for Biotechnology Information (NCBI; https://www.ncbi.nlm.nih.gov/) supplies taxonomic and genomic datasets. Phylogenetic information is increasingly supported through TreeBASE (https://www.treebase.org/treebase-web/home.html), and species trait and metacommunity datasets are available through CESTE (https://icestes.github.io/). Climate and environmental predictors are widely derived from WorldClim (https://www.worldclim.org/data/bioclim.html). In ecological interaction and network-based studies, platforms such as Mangal (https://mangal.io/#/) and Global Biotic Interactions (GloBI; https://www.globalbioticinteractions.org/about) are increasingly used for realised and potential species interaction datasets. The growing interoperability of these databases supports integrated machine learning, ecological forecasting and spatial prediction approaches for biodiversity and invasive species monitoring.

Given these impacts, advanced remote sensing methods are needed for early detection, spatial risk assessment and control (Table 1). Table 1 summarises the positive and negative impacts reported across 149 studies, with the corresponding references listed in Appendix A of the supplementary material (https://www.scidb.cn/en/anonymous/WkYzZVly). Because a study could report more than one impact, impact category counts are not mutually exclusive. This review evaluates the strengths, limitations and future prospects of remote sensing technologies for invasive plant management.

**Table 1:** Positive and negative impacts identified using remote sensing, with supporting references

Sl no.	Continent	No. of research studies	Positive impacts			Negative impacts					References^*^
	Region		Medicinal	Aesthetic	Climate	Agricultural loss	Diseases	Forest degradation	Endangered threat	Water depletion	
1	Africa	20	Two	Three	Two	Six	Zero	Three	Four	Five	26,30,31,32,36,52,54,57,62,63, 78,86,99,101,112,115,122,126, 138,145
2	Asia	25	Seven	Five	One	Seven	Two	Seven	Fourteen	Two	5,6,13,20,74,77,116,121,128, 132,141,142,144,146,147,148, 149,150,151,152,153,154, 155,156,157
3	Australia	4	Zero	Zero	Zero	One	Zero	One	Two	Zero	2, 51, 53, 163
4	Europe	22	Two	Four	Four	Six	One	Zero	Two	Two	3,9,17,27,28,29,41,46,59,71, 72,75,85,92,94,95,96,100, 103,108,110,111
5	Global	27	Two	Four	Zero	Two	Three	Three	Nine	Four	10,11,12,18, 24,34,37,38,43,45, 47,49,58,60,61,65,67,69,80, 81,82,85,88,98,102,104,105
6	North America	42	Zero	Five	One	Five	Zero	One	Six	Five	4,8,14,19,21,23,25,33,35,39, 40,42,44,48,55,56,64,68,70,76, 79,83,87,93,106,107,109,113, 117,118,120,127,131,134,135, 136,158,159,161,162, 164,166
7	South America	7	Two	One	Zero	Zero	One	Two	One	Zero	15,16,22,50,66,89,97
8	Tropical	2	Zero	Zero	Zero	Zero	Zero	One	One	Zero	73,91
	**Total**	**149**	**15**	**22**	**8**	**27**	**7**	**18**	**39**	**18**	

*The supplementary data with all the references used are provided in Appendix A of the supplementary information (https://www.scidb.cn/en/anonymous/WkYzZVly)

Previous reviews of remote sensing for invasive species have generally emphasised particular sensors, regions or methodological advances. This review differs by integrating bibliometric analysis, 25-year publication trends, the evolution of sensors and platforms, predictive modelling and machine learning, reported classification accuracy, global research disparities and linkages with the United Nations Sustainable Development Goals (SDGs). This combined perspective connects technological development with ecological applications and policy relevance, providing a consolidated account of progress and remaining needs in remote sensing–based invasive plant monitoring.

## Methodology

To analyse the global trajectory of remote sensing applications in invasive plant research, the review used four sequential stages: database search and record export, duplicate removal and relevance screening, full-text eligibility assessment and evidence coding with quantitative literature synthesis. Web of Science and Scopus were searched using Boolean combinations of (“invasive plant species” or “invasive species” or “biological invasion”), (“remote sensing” or “satellite imagery” or “hyperspectral” or “multispectral” or “synthetic aperture radar” or “light detection and ranging”) and (“mapping” or “detection” or “monitoring”). Each eligible study was assigned to the smallest geographic unit that encompassed its study area; studies explicitly focused on India or China were retained as separate regional categories to avoid overlap.

Studies were included when they focused on invasive plant species, applied remote sensing methods and reported quantitative or spatial results in a peer-reviewed journal article. Studies were excluded when they focused on nonplant invasive species, did not use remote sensing, lacked sufficient methodological detail or were reviews, conference abstracts or reports without full datasets. Screening proceeded from duplicate removal and title, abstract and keyword review to full-text eligibility assessment.

**Fig. 1:**
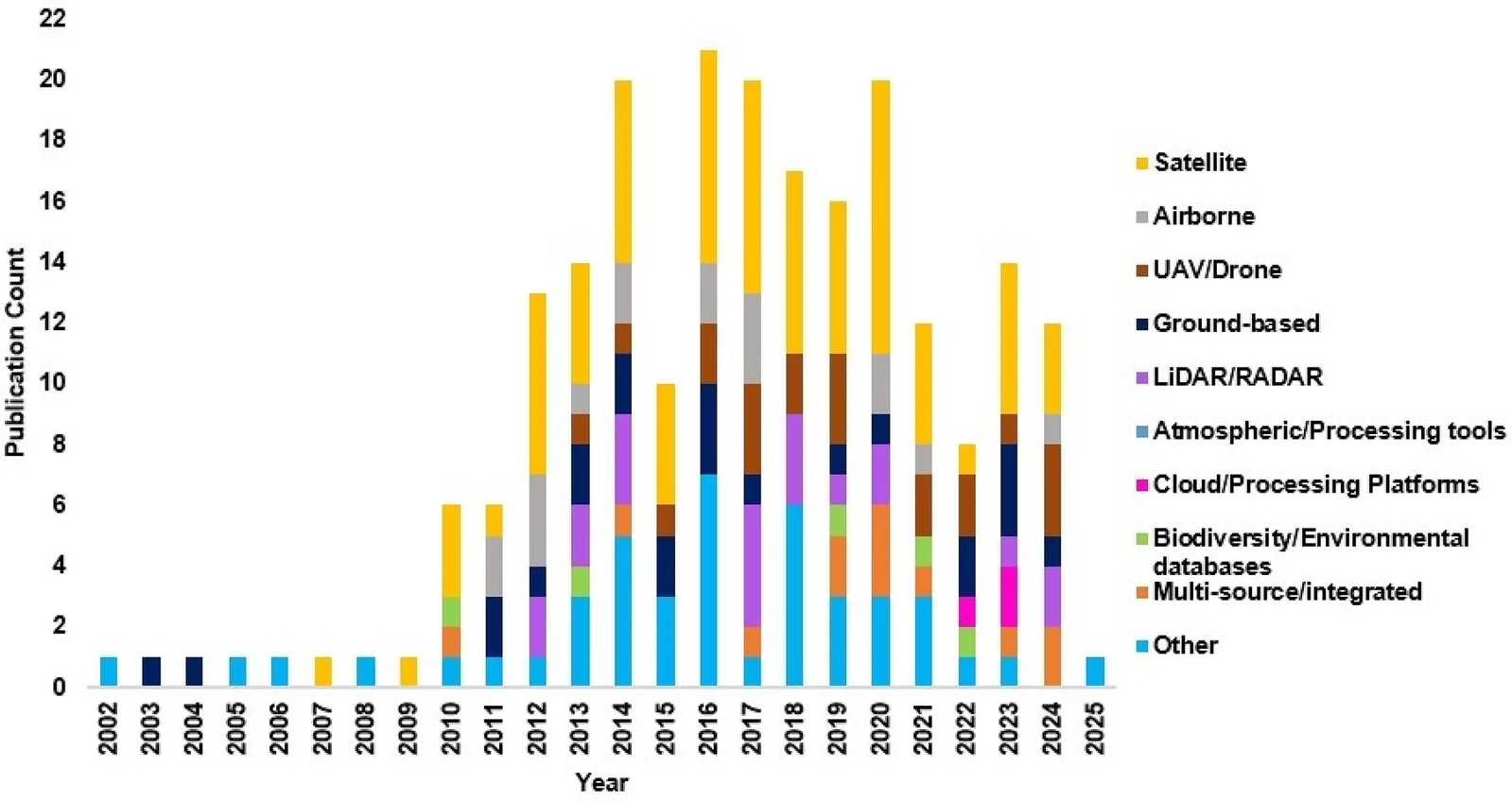
Publication counts per year and dataset or sensor used

The database export contained 411 bibliographic records. After screening, 166 primary studies were retained for detailed review and are listed in Supplementary Tables S1 and S2. This core set supported the temporal, regional, sensor and methodological analyses. Within the 166 studies, 149 contained extractable information on positive or negative impacts and were summarised in Table 1, while 76 explicitly reported overall accuracy values and formed the accuracy-analysis subset shown in Fig. 1. The values 411, 166, 149 and 76 therefore represent successive or purpose-specific analytical sets rather than conflicting totals. Supporting study-level references are provided in Appendix A.

**Fig. 2:**
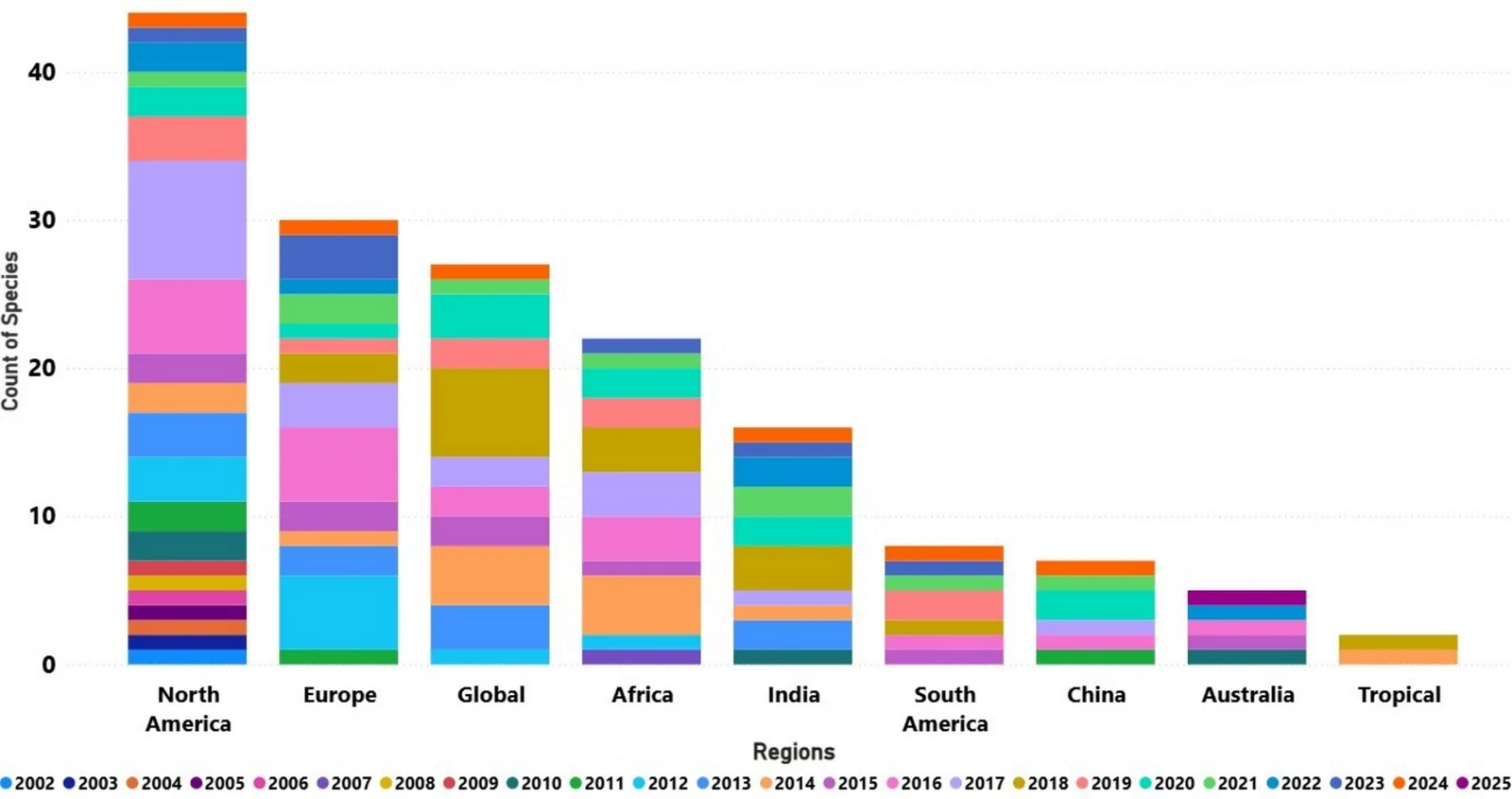
Temporal and regional distribution of invasive plant species studies from 2002 to 2025

**Fig. 3:**
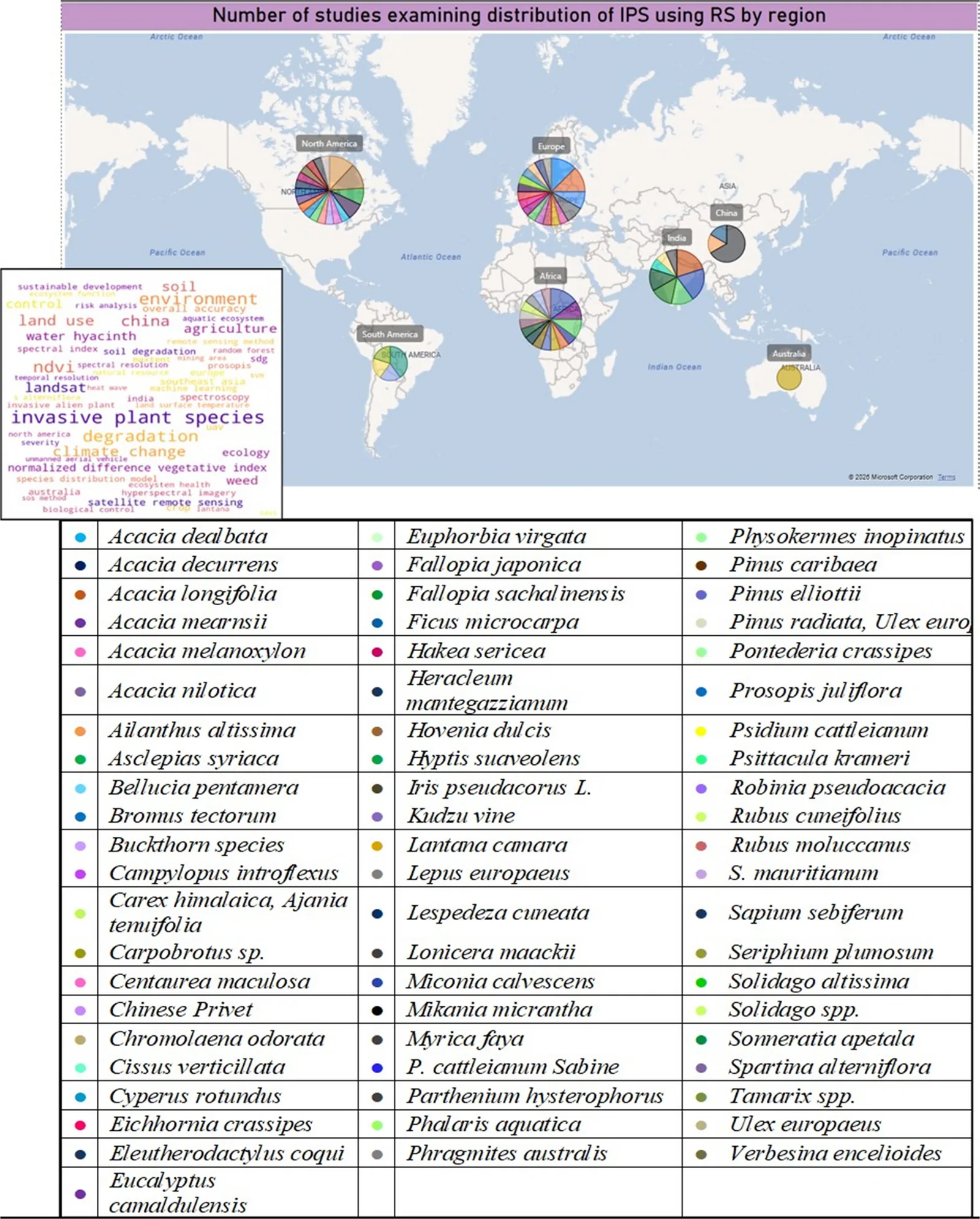
Global distribution of invasive plant species studies by region; pie size represents the number of studies and coloured segments represent species composition (top). Word cloud showing the relative frequency of terms used in remote sensing-based invasive plant research (bottom)

Temporal publication trends were examined through a quantitative literature synthesis. Microsoft Excel was used for data acquisition, cleaning and categorisation, and Power BI was used for visualisation. Studies published from 2000 to 2025 were classified by remote sensing platform and dataset or sensor type, including optical, multispectral, hyperspectral and synthetic aperture radar data. Annual publication counts and sensor use were displayed as stacked bar charts. Bibliometric analysis was used to examine keyword relationships and thematic development. The full export of 411 bibliographic records was imported from EndNote in RIS format into VOSviewer for full-counting co-occurrence analysis. Of 574 keywords, 11 met the minimum threshold of five occurrences and were included in the network map shown in Fig. 2. Recurring terms were also visualised as the word cloud included in Fig. 3 using Python, the wordcloud library and matplotlib. The results of this workflow are presented below as temporal, regional, bibliometric, accuracy and technological trends.

## Results

From 2000 to 2025, the number of studies using remote sensing for invasive plant monitoring increased markedly. This growth coincided with the expansion of uncrewed aerial systems, cloud computing platforms (Singh et al., 2020), machine learning methods (Ahmed et al. 2021; Jensen et al., 2020) and satellite missions offering improved combinations of spatial, temporal and spectral resolution (Lake et al., 2022; Rivas-Fandiño et al., 2023). The evidence base is now approximately 20 times larger than in the early 2000s. Airborne research expanded more slowly than satellite- and uncrewed aerial system-based research, with the latter increasing rapidly after 2012 (Fig. 1). Figure 1 shows annual publication trends, while Table 2 summarises sensor use in the literature for the platform categories derived from the datasets and sensors reported in each study. Categories include satellite, airborne, uncrewed aerial systems or drones, ground-based systems, light detection and ranging, radar, atmospheric or processing tools, cloud processing platforms, biodiversity or environmental databases, multi-source or integrated data and other sources. A study could be assigned to more than one platform category when multiple datasets were used (Appendix A).

**Table 2:** Sensors used over the last 25 years, corresponding to Fig. 1

Platform category	Platform/data source	Sensor type	Key reference/methods
Satellite	Landsat (MSS, TM, ETM +, OLI, Landsat 5, 7, 8)	Multispectral	GLMs, GAMs, MaxEnt, GARP, DNN, BSVM
	Sentinel	Multispectral	Integrated modelling approaches
	MODIS		Climate and vegetation analysis
	IRS (LISS-III, LISS-IV, P6), Cartosat- 1	Multispectral/Thermal	Land-use and land-cover analysis
	EO-1 (ALI, Hyperion)	Multispectral	Fine-scale mapping, biodiversity assessment
	Worldview-2, QuickBird, Pleiades, RapidEye, CEBERS-4A	Multispectral/Hyperspectral	Vegetation indices, spectral analysis
Airborne	AVIRIS, APEX, HyperSpecTIR	Hyperspectral	Vegetation indices, spectral analysis
	Airborne imaging Spectroradiometer	Hyperspectral	Environmental monitoring
	Airborne orthophotos, aerial photographs	Optical/RGB	Visual interpretation, mapping
UAV/drone	UAV-based imagery	Optical/RGB (high-resolution)	Field validation fine-scale mapping
Ground-based			Terrain and spatial analysis
	ASD Field Spec (3,4 Hi-Res, Standard, FR)	Spectroradiometer (VNIR-SWIR)	Field spectroscopy, calibration/validation
	(3, 4 Hi-Res, Standard, FR)	Spectroradiometer (VNIR-SWIR)	Structural analysis, canopy height and terrain modelling
	GNSS, DEM	Elevation/Topographic	Terrain and spatial analysis
	Field observations, ground truth data	_	Validation and accuracy assessment
LiDAR/RADAR	LiDAR datasets, RADAR datasets	Active remote sensing	Structural analysis, canopy height, terrain modelling
Atmospheric/processing tools	MODTRAN LUT, ACRON model	Atmospheric correction	Pre-processing of hyperspectral data
Cloud/processing platforms	Google Earth Engine (GEE)	_	Large-scale geospatial analysis and computation
Biodiversity/environmental databases	GBIF, global biodiversity databases	_	Species occurrence and distribution data
	BioCLIM, BRUTO datasets	_	Ecological Niche Modelling
	Climate datasets (hourly), vapour pressure deficit	_	Environmental variable inputs
Multisource/integrated	Combined datasets (Landsat, Sentinel, MODIS, LiDAR)	Multisensor	Integrated modelling and landscape-level analysis
Other	Literature review	_	Synthesis of existing knowledge

**Fig. 4:**
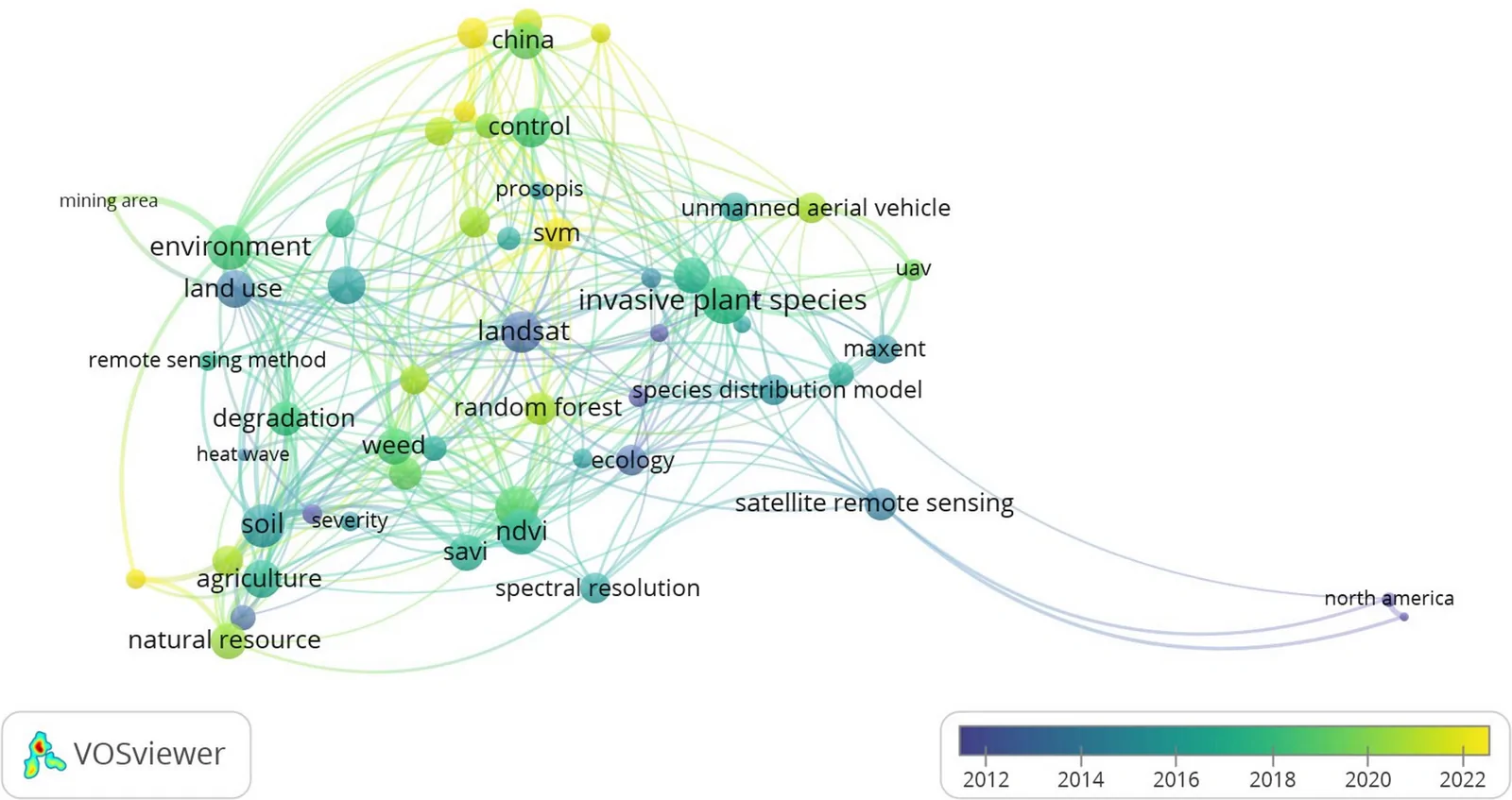
Keyword co-occurrence network generated in VOSviewer for invasive species research. Node size indicates occurrence frequency, links indicate keyword co-occurrence, and colour represents average publication year from earlier studies in blue to more recent studies in yellow

The stacked bars in Fig. 4 show that satellite datasets dominate across the review period, while uncrewed aerial systems, light detection and ranging and radar increase in recent years. Airborne, ground-based and cloud processing platforms occur less frequently but demonstrate growing methodological diversity.

**Fig. 5:**
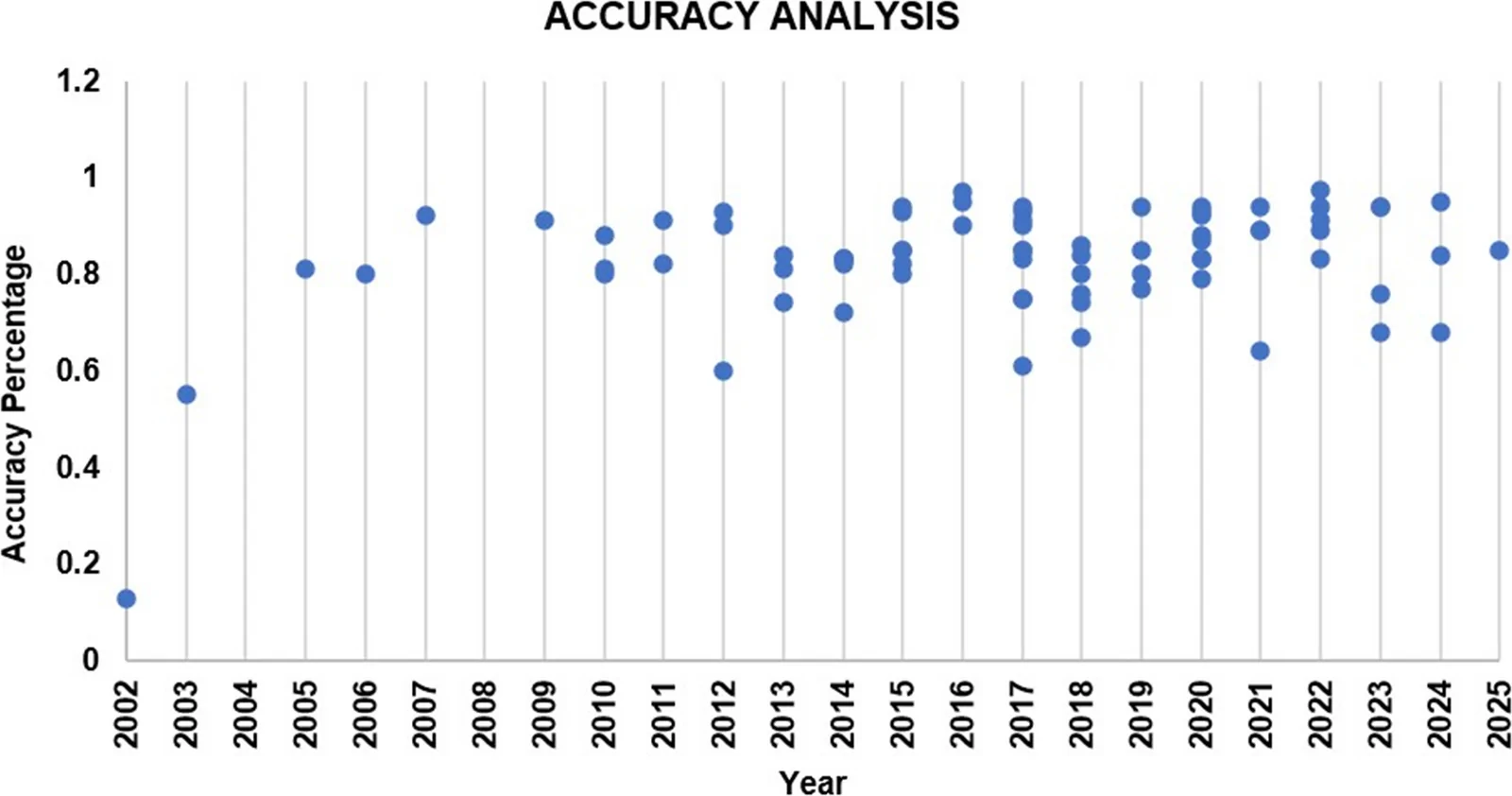
Reported overall accuracy values in remote sensing-based invasive species studies. Values are study-specific and are not directly comparable across sensors, regions, classification methods, and validation designs

Figure 5 presents regional and temporal variation in study occurrences across Africa, India, South America, China, Australia, North America, Europe and the global and tropical categories. India and China were retained as distinct units when studies explicitly reported them separately. In this analysis, *n* denotes the number of study occurrences recorded for a region and year, not the number of unique species worldwide. A species may therefore contribute more than once when reported in different studies, regions or years. North America has the largest number of study occurrences, with more than 40 records overall and substantial activity between 2006 and 2014. Europe and the global category follow with approximately 30 and 27 records, respectively. Africa and India show moderate representation, with approximately 22 and 17 records. South America, China, Australia and tropical regions remain less represented, although activity in South America and China increased between 2018 and 2022. Studies classified as tropical appear mainly in 2023–2025, indicating recent attention to these biodiversity-rich regions.

From 2010 to 2013, the primary objective of IPS research was to monitor extensive vegetation areas, with a particular emphasis on satellite platforms such as Landsat. A substantial increase in the use of terms such as “spectral resolution,” “North America” and “satellite remote sensing” occurred between 2012 and 2016. During this period, research activities centred on regional mapping and sensor evaluation. The emergence of terms such as “NDVI”, “random forest” and “species distribution models” indicates that supervised classification and vegetation indices were implemented at an early stage. Following 2015, an increasing number of studies implemented real-time detection and machine learning. Terms such as “control,” “unmanned aerial vehicle,” “deep learning” and “SVM” became more common from 2018 to 2022.

Subcanopy IPS in tropical rainforest have been detected by integrating optical and microwave (InSAR/PolInSAR) RS data, with a decision tree algorithm sensor (Ghulam et al., 2014). The majority of studies conducted after 2016 (Fig. 4) have concentrated on optical, multispectral and hyperspectral sensors (detailed sensor assessment is shown in Table 2). Interestingly, hyperspectral sensors (He et al., 2011; Mansour, 2012) have become more popular despite their high cost and demanding processing requirements

Advances in sensor capability and spectral discrimination have improved invasive plant detection. Carter et al. (2009) compared summer-acquired Hyperion, Landsat Thematic Mapper and QuickBird data for mapping tamarisk along the Colorado River. Dai et al. (2020) combined field observations and remotely sensed data to map understory invasive plants, including *Mikania micrantha*, in Chitwan, Nepal. Diao and Wang (2014) developed a fine-resolution remote sensing model for invasive-species distribution. These studies illustrate how spatial resolution, field calibration and species-specific spectral or phenological information affect mapping performance.

Network-based bibliometric analysis further highlights evolving research themes. Network visualisations (Fig. 2) indicate that “invasive plant species” is the most prominent keyword, alongside other important methodological keywords. Thematic nodes including “soil”, “agriculture”, “land use” and “degradation” illustrate the practical applications of IPS research across various disciplines, including ecology, agriculture and land management.

As shown in the network visualisation diagram (Fig. 2), the keyword “invasive plant species” is the most dominant node, with the highest co-occurrence value (55), total link strength (80) and cluster strength (10).

Seasonal detection limitations, interference from vegetation structure and selection of appropriate wavelengths are among the challenges in RS-based species identification (Burkholder et al., 2011). However, multispectral (Becker et al., 2013) and hyperspectral (Somers & Asner, 2013b) analyses based on leaf chemistry and leaf water content have improved detection precision.

For example, AVIRIS imagery was utilised to identify *Carpobrotus edulis* and *Cortaderia jubata* in California, partly because of their higher leaf water content.

## Discussion

The worldwide distribution of IPS is influenced by a complex interplay of life history traits, climate, human intervention and data availability (Chapman et al., 2017). It is vital to map current IPS distributions and identify the environmental variables that facilitate their expansion. As plants develop and their form and function change, their niche requirements may also change (Gabler & Siemann, 2012). The regeneration niche is the set of characteristics that are needed for early growth. These requirements often differ from those of mature life stages (Mologni et al., 2022; Sotomayor & Lortie, 2015). Juvenile IPS often have narrower tolerance ranges, particularly under stressful conditions; therefore, reliance on mature distributions alone can overestimate potential spread (Walck et al., 2011). It is also important to distinguish naturally occurring species from those planted or supported by humans, because assisted establishment can inflate estimates of suitable habitat (Sax et al., 2022). Reliable assessments therefore require carefully curated occurrence data to reduce bias in distribution modelling. These methods and sensors can recognise distinct structural and biochemical traits (Gupta et al., 2022a, 2022b) of single species, including IPS, within mixed-canopy signals. For consistency with the analytical framework, broader continental categories such as Asia were not used where country-specific analyses (e.g., India and China) were explicitly available, and conversely, country-level classifications (India and China) were not included where studies were grouped under Asia, ensuring nonoverlapping regional classification.

In addition to technical advancements, bibliometric analysis reveals how conservation and restoration agendas are converging within IPS research. As severe ecosystem degradation follows rapid industrialisation (Sharma et al., 2022), ecological restoration is gaining traction worldwide as a response to biodiversity loss and land degradation. This indicates a progression towards enhanced automation and precision in IPS detection systems.

Despite the ecological significance of such analyses, insufficient data distribution and limited access has long constrained global-scale assessments of IPS. Despite the theoretical foundations provided by macroecological assessments of local floras (Pyšek et al., 2017), vascular plant datasets have historically had a smaller spatial coverage than those for mammals, reptiles, molluscs, birds and bryophytes (Essl et al., 2015; Pyšek et al., 2020). Research on invasion pathways and biogeographic patterns has been hampered by this data gap (Pyšek et al., 2017). Recent initiatives and major biodiversity and environmental databases now support comparisons across continents, including the BONAP database (https://bonap.net/TDC/CGD) (Narango et al., 2020), the EPPO global database (Arslan et al., 2015; Brunel et al., 2010) (https://www.eppo.int/activities/iap_activities), Indian Alien Flora Information Database (ILORA) (https://ilora2020.wixsite.com/ilora2020) (Pant et al., 2021; Prajapati et al., 2022), the National Invasive Species Information Center (NISIC) database (https://www.invasivespeciesinfo.gov/) (Corn & Johnson, 2013) and the DAISIE project (https://europe-aliens.org/) (Kalusová et al., 2024; Simberloff & Rejmánek, 2011), which have collectively improved data availability (Dawson et al., 2013; Van Kleunen et al., 2015) and access to occurrence, climate and distribution records. Although they facilitate more comprehensive analyses, international compilations such as Randall’s Compendium (2002, 2012) are still constrained by inconsistent data quality and a lack of standardisation (Dawson et al., 2013). Regional differences in monitoring intensity, taxonomic resolution and data availability still exist (Kalusová et al., 2024; Narango et al., 2020). The United States shows strong international collaboration, whereas China surpasses in publishing volume.

This imbalanced trend is a reflection of historical differences in funding availability, research infrastructure (Rusňák et al., 2022) and institutional emphasis (Mallmann et al., 2023) on ecological monitoring (Martinez et al., 2020). Due in large part to their early adoption of cutting-edge RS technologies, longstanding academic investment in ecological research and strong policy frameworks (Stoett et al., 2019) targeting IPS, North America and Europe have dominated the field. For example, regional emphases differ—afforestation in the U.S., grassland recovery in Oceania and post-industrial rehabilitation in Europe (FU Zhanyong et al., 2019), but each intersects with patterns of IPS propagation

Despite advances in remote sensing, regional disparities persist: Africa, Asia and South America remain underexplored (Fuentes et al., 2013; Henderson, 2011), while Europe is overrepresented because of early inventory efforts (Simberloff & Rejmánek, 2011). Data coverage and technical capacity remain limited in many biodiversity-rich regions. Climate, anthropogenic factors and life history traits all influence patterns of global distribution (Valladares et al., 2016). It is still imperative to address gaps in data coverage, standardisation and monitoring of IPS (Dawson et al., 2013; Pyšek et al., 2017). Formulating equitable and universally applicable invasion management strategies necessitates the resolution of these discrepancies (Van Kleunen et al., 2015).

Research activity has also increased in parts of the Global South, including India, China and Africa, reflecting growing recognition of invasive plant threats and improving access to open remote sensing data and machine learning tools. Nevertheless, limited funding, restricted access to high-resolution data and uneven technical capacity continue to constrain research in many tropical and developing regions (Ahmed 2020; Zaka & Samat, 2024).

Regional differences are evident among the 168 regional study occurrences recorded from 2002 to 2024. North America accounts for 26.19% of study occurrences (Milledge et al., 2016; Sladonja et al., 2015), followed by Europe (17.86%) and global studies (16.07%) (Halmy 2024; Rodriguez-Garlito et al., 2023). Australia and tropical regions account for 2.98% and 1.19%, respectively (Bradshaw et al., 2021; Ghulam, 2014), while Africa contributes 13.10% (Cuneo et al., 2009). Satellite methods are used widely because of their accessibility, whereas uncrewed aerial and airborne approaches remain more resource-intensive.

Hyperspectral data are reported predominantly in North American and European studies. In the sensor-specific subsets reported in Table S1, North America accounts for 32.00% of Landsat studies and 25.00% of Sentinel-2 studies. In countries such as China and South Africa, researchers frequently utilise multispectral data from Sentinel and Landsat satellites to map and monitor changes in vegetation cover, assess the spread of invasive species and manage agricultural landscapes on a regional scale. However, some satellite-based studies do not specify the sensors used. Incomplete sensor reporting may bias evaluation of sensor-specific trends.

In IPS research, word clouds (Fig. 3) produced through bibliometric analyses are useful resources that highlight the frequency of keywords extracted from dominant themes, commonly researched species and methodological approaches. Frequently occurring terms include “invasive plant species”, “remote sensing” and “NDVI,” indicating a strong focus on vegetation monitoring. The frequent occurrence of vegetation indices such as NDVI and SAVI reflects their widespread use as assessment indicators in remote sensing-based studies. The terms “invasive plant species”, “NDVI”, “China”, “degradation”, “climate change” and “Landsat” are prominent. Terms that are less frequently used, such as “SAVI”, “heat wave” and “unmanned aerial vehicle” indicate areas of methodological innovation or growing interest. While Fridley (2008) and Richardson and Pyšek (2012) emphasise the recognition of taxonomic and geographic patterns in invasion ecology, Pyšek et al. (2009) and Dawson et al. (2013) recommend looking at keyword and species frequencies to identify regional differences in research focus. Also, as Fig. 3 shows, the Global South, especially Africa, India, China and tropical regions, has shown a marked increase in research activity only in recent years, likely driven by both escalating ecological pressures and growing accessibility to open-source RS platforms like Landsat and Sentinel (Mallmann et al., 2023; Rusňák et al., 2022).

Beyond keyword patterns, species- and region-based distributions further highlight research focus areas (Jose et al., 2013). Future prospects include thermal and mid-infrared emissivity spectra research and sensor development (Harrison et al., 2018), with studies like Burkholder et al. (2011) emphasising the importance of band and timing selection for accurate mapping of species such as *Ailanthus altissima*. These innovations have elevated RS into a globally scalable tool, influencing both temporal and spatial research directions.

## Improved prediction accuracy through technological advancement

Accuracy assessment is a critical post-classification validation technique that ensures the reliability and precision of the plant species invasion detection models (Sabat-T. et al., 2020). In remote sensing applications for monitoring IPS, accuracy assessment provides a basis for dependable data interpretation and informed decision-making. The reliability of classification maps in detecting IPS is directly associated with accuracy metrics, including user accuracy, producer accuracy and overall accuracy (OA). Additional metrics like *F*1-score, area under coverage (AUC) and true skill statistic (TSS) are also utilised. These metrics evaluate the performance of an RS model and help determine the applicability of the results in ecological management and policy interventions. Figure 1 summarises overall accuracy values reported by 76 studies. Values were extracted as reported by the original authors and were not averaged across datasets or sensor types. Because sensor characteristics, spatial resolution, classification methods, training data and validation designs differ among studies, the points should be interpreted as study-specific results rather than directly comparable benchmarks.

Across the reviewed studies, OA values generally range from approximately 60% to 95%. Within this heterogeneous evidence base, lower accuracy values tend to be associated with coarse-resolution datasets, heterogeneous landscapes and early classification approaches, while higher accuracies tend to be reported in studies using hyperspectral data, UAV-based imagery and advanced machine learning techniques. These accuracy values should be interpreted as study-specific performance indicators rather than directly comparable benchmarks across studies.

In the early 2000 s, reported overall accuracy was often approximately 60–70% because of coarse-resolution data, basic classification techniques and reliance on low-resolution satellite imagery, such as MODIS and Landsat 1–3 missions (Hunt et al., 2003; Karnieli et al., 2013). The restricted availability of sensors and the limited geographic scope of the studies constrained the depth of analysis.

The availability of open-access data from Sentinel-2 (Dai et al., 2020), MODIS (Rodriguez-Garlito et al., 2023) and UAV (Ojija et al., 2024) improves temporal coverage and enables targeted monitoring through time-series analysis. Because IPS often display phenological, structural and spectral heterogeneity across regions and seasons, periodic validation is needed to assess the geographic and temporal reliability of classification outcomes (Müllerová et al., 2013; Pyšek et al., 2017). Mapping studies of Spiraea tomentosa in Poland, utilising HySpex imagery, demonstrated *F*1-score accuracies reaching 83% and an overall accuracy of 99% (Sabat-Tomala et al., 2022). This example shows how temporal analysis and sensor selection can support high-quality mapping.

Ensemble and artificial intelligence–based classification can reduce misclassification in heterogeneous vegetation. Recent studies also test emerging algorithms and sensors in underrepresented regions where cloud cover, mixed pixels and structural complexity complicate detection. Repeated training and validation can quantify classification stability and support probability-based maps, which are particularly useful for early infestations and species with subtle spectral signatures.

The methodological choices described above directly influence mapping reliability and ecological applicability in invasive species studies. Precise vegetation mapping is essential for timely interventions against invasive species and for ecological management. Inaccurate outputs might obstruct efforts to mitigate the effects of harmful invaders, such as *Heracleum mantegazzianum*, which poses health and ecological risks due to its dermal toxic compounds (Nasadiuk & Mamchur, 2024). Precise maps enhance operational monitoring, targeted restoration and effective resource distribution. Dai et al. (Dai et al., 2020) have shown that VHR images markedly enhance the landscape-level mapping of *Lantana camara* in Indian forest reserves. Ecological modelling was first focused on data-abundant regions, specifically North America and Europe. However, between 2010 and 2020, it expanded to include inputs from China, India and Africa. The appearance of aggressive species such as *Prosopis juliflora* and *Parthenium hysterophorus* in biodiverse zones underscores the need for localised detection models (Nandasena et al., 2023; Ojija et al., 2024). These are further refined using hyperspectral data and field validation. Ecological urgency can stimulate iterative refinement of predictive-mapping techniques, improving their accuracy and spatial coverage. These application-driven insights further reinforce the importance of ensemble methods, hyperspectral data and localised model calibration discussed above.

Early adopters like North America and Europe achieved consistent results due to strong satellite infrastructure and data-sharing platforms, unlike under-resourced regions that entered later (Dai et al., 2020; Ojija et al., 2024). Reported gains in accuracy are associated with advances in sensor technologies and algorithmic sophistication, responding to the growing ecological footprint of IPS, such as *Prosopis juliflora* and *Lantana camara*. Rather than being a final validation step, accuracy assessments now operate as essential feedback loops that refine model inputs and strengthen semi-automated detection systems. These validated metrics can inform policymakers and forest departments when selecting interventions, whether mechanical, chemical or biological, and support monitoring frameworks that are transparent, scalable and ecologically sound. The integration of accurate classification supports intervention-ready mapping and long-term ecosystem resilience.

In essence, classification accuracy (Mallmann et al., 2023; Rodriguez-Garlito et al., 2023) underscores the intersection of technological (Dai et al., 2020; Vaz et al., 2018) readiness, ecological awareness (Witt et al., 2018) and regional response capacity. IPS persists in destabilising ecosystems due to climate change (Mallmann et al., 2023; Underwood et al., 2003) and intensified land use (Mallmann et al., 2023; Tadros et al., 2020), necessitating high-accuracy models for early detection, policy development and restoration strategies. This highlights the need for standardised validation frameworks in future studies to enable more robust and comparable accuracy assessments.

## Linkages between invasive species impacts and the SDGs

Many studies over the past few years have identified the interconnections between IPS and the United Nations SDGs (Hussien et al., 2023; Mqingwana et al., 2024; Saxena et al., 2024), using a quasi-quantitative approach (Bimrah et al., 2022; Mitra et al., 2024) based on the observed ecological impacts (Witt et al., 2018; Shackleton et al., 2014; Halmy 2024), RS techniques and global response frameworks (Bradley et al., 2010; Stoett et al., 2019). In particular, the goals about the biosphere, which form the cornerstones of sustainable economies, societal well-being and ecological resilience, make tackling biological incursions intrinsically tied to the SDG mission of advancing a shared global vision for sustainability.

**Fig. 6:**
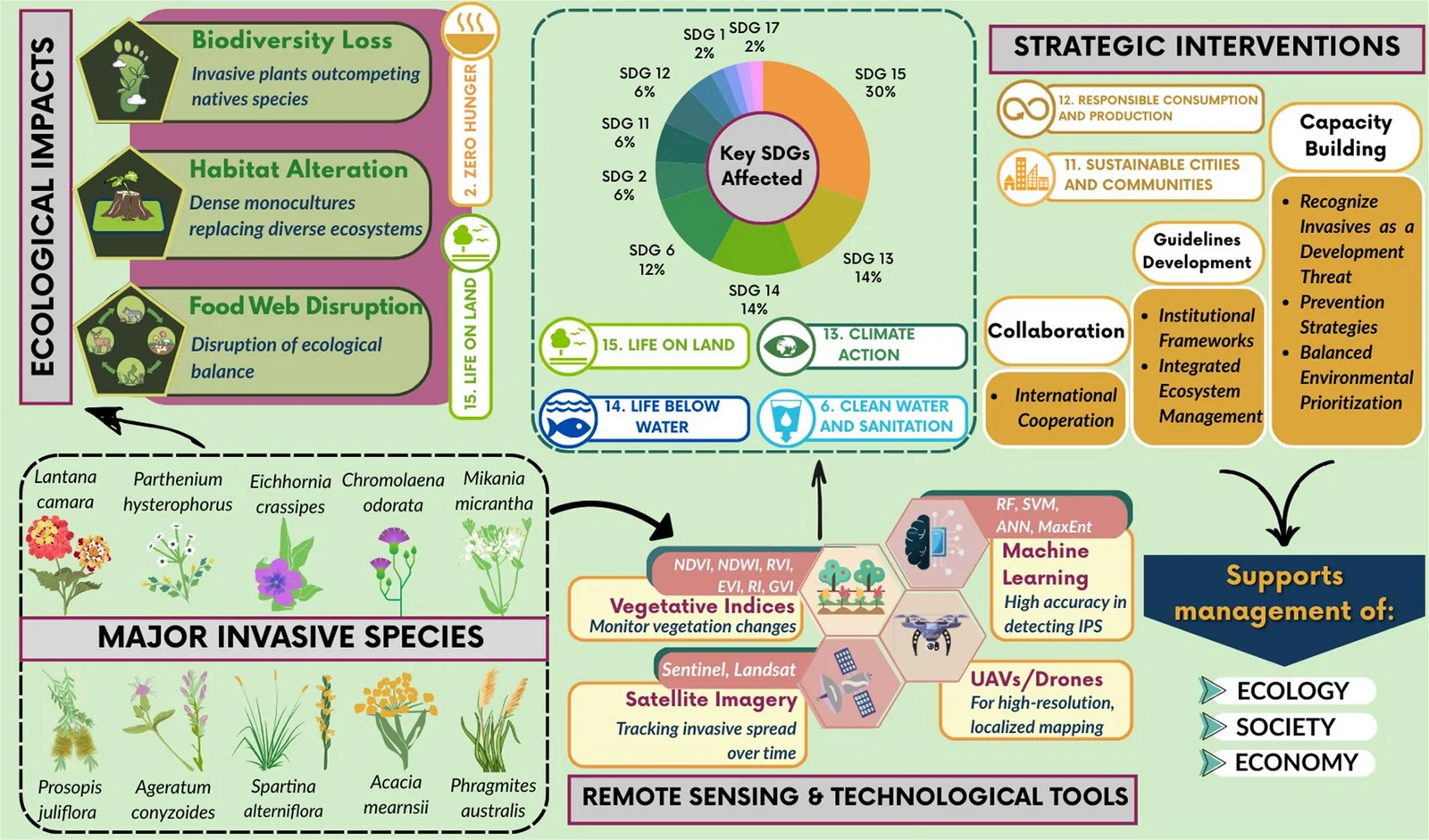
Integrated framework linking invasive plant species, ecological impacts, and remote sensing-based assessment methods with relevant Sustainable Development Goals and management interventions

To assess the linkage between IPS-related studies and the United Nations Sustainable Development Goals (SDGs), each publication was manually evaluated based on its study objectives, thematic focus and reported outcomes. SDGs were assigned by aligning the study content with relevant SDG targets, such as ecosystem conservation (SDG 15), water resource management (SDG 6) and climate-related impacts (SDG 13). In cases where studies addressed multiple thematic areas, more than one SDG was assigned to a single study. The SDG-wise distribution of IPS-related publications was assessed and represented in the visualisation. Data acquisition and preprocessing were performed using Microsoft Excel. The relevant subset of publications linked with the SDGs was extracted from the table (available in Appendix A). Numerical frequency counts were compiled for each SDG, converted to percentages of total assignments, and the resulting distribution was visualised through a pie chart. This approach enabled quantification of publication alignment with different SDGs, thus informing the distribution pattern reflected in the figure. The summarised data visualisation is presented in the pie chart in Fig. 6.

Of the total 166 reviewed publications, only 21 studies explicitly addressed linkages between invasive plant species (IPS) and the United Nations Sustainable Development Goals (SDGs). These studies were used to assess SDG-wise distribution. As individual studies often addressed multiple thematic dimensions, a single study could be assigned to more than one SDG category, resulting in overlapping counts. Therefore, the reported counts and percentages are based on total SDG assignments rather than the number of unique studies, so the sum of category counts can exceed the 21 unique studies. Across these assignments, SDG 15 (Life on Land) was assigned in 15 studies, followed by SDG 13 (Climate Action) and SDG 14 (Life Below Water), each represented by 7 studies. SDG 6 (Clean Water and Sanitation) was associated with 6 studies. Similarly, SDG 11 (Sustainable Cities and Communities) and SDG 12 (Responsible Consumption and Production) were each represented by 3 studies. Other SDGs, such as SDG 2 (Zero Hunger) and SDG 1 (No Poverty), with counts of 3 and 1, respectively, were indirectly linked through their association with ecosystem services, land productivity and food security.

This review shows that IPS management is mainly connected to nine SDGs, even though some contribute more than others. According to the distribution in Fig. 6, SDG 15 (Life on Land) (Saxena et al., 2024; Waseem & Halmy, 2024) has the highest alignment, accounting for 30% of the total SDG assignments. This reflects the detrimental effects of invasive plants on ecosystem structure, terrestrial habitat degradation and native biodiversity. Numerous studies have shown how invasive monocultures damage endangered plants and animals, alter food webs and replace a variety of natural habitats. Similarly, SDG 13 (Climate Action) (Halmy 2024; Bimrah et al., 2022) and SDG 14 (Life Below Water) (Bimrah et al., 2022; Chen & Yen, 2023) each account for 14% of the contributions. IPS can impair carbon storage capacity, aggravate land degradation and disturb hydrological and coastal systems, which intensifies vulnerability to climate-related catastrophes. IPS management can help countries meet their Nationally Determined Contributions (NDCs) under the Paris Agreement and is in line with international climate restoration initiatives like the UN Decade on Ecosystem Restoration. With 12%, SDG 6 (Clean Water and Sanitation) (Mqingwana et al., 2024) is also extremely important because of the effects of IPS on water bodies and wetland ecosystems, especially through organisms like *Eichhornia crassipes* that lower aquatic oxygen levels and deteriorate water quality.

Additionally, SDG 11 (Ekka et al., 2023; Garzon-Lopez et al., 2024; Persello et al., 2022) and SDG 12 (Garzon-Lopez et al., 2024; Jensen et al., 2020; Vaz et al., 2019) each account for approximately 6%. The emergence of invasive plant species is largely influenced by the encroachment of urban development into natural ecosystems and the prevalence of unsustainable land-use methods. Deforestation, agriculture, aquaculture and infrastructure development, including ports and power plants, play a significant role in the spread of invasive species. These activities can negatively affect ecosystems and conflict with the principles of sustainable resource management. Other Sustainable Development Goals, such as SDG 2 (Zero Hunger) (Halmy et al., 2015; Ojija et al., 2024) and SDG 1 (No Poverty) (Ojija et al., 2024), are indirectly associated with these objectives due to their connections to food security, local livelihoods and land productivity. The dominance of IPS over cropland and grazing systems is increasingly impacting these areas.

Despite the significance of these links, current remote sensing-based IPS studies seldom examine connections with numerous SDGs, particularly those pertaining to health, education and gender equality. This suggests that future research should adopt a more integrated, cross-sectoral approach.

## Role of RS in studying IPS case studies

Remote sensing supports invasive plant detection and monitoring across a range of spatial scales. Satellite sensors, airborne hyperspectral systems, light detection and ranging, radar and uncrewed aerial systems can characterise spectral, structural, moisture and phenological differences. Their value is greatest when combined with field observations, machine learning and habitat modelling.

### Satellite and airborne data sources and sensors

Airborne and satellite-based remote sensing platforms provide multiple tools for mapping IPS. These tools depend on the spatial scale, sensor type and spectral range. UAVs, or drones, have become increasingly common because they are flexible, collect high-resolution imagery and can be relatively cost-effective (Kattenborn et al., 2019). UAVs were initially developed for military use, but now are utilised in ecological research and carry RGB, multispectral, hyperspectral, thermal and LiDAR capabilities (Colomina & Molina, 2014). When used with object-based image analysis (OBIA) and machine learning (Tmušić et al., 2020; Zaka & Samat, 2024), these sensors can help distinguish phenological and structural differences between invasive and native species. UAVs also help with hard-to-reach areas and support fine-scale validation (Hugenholtz et al., 2013; Müllerová et al., 2017).

Multispectral satellite systems such as Landsat 8 Operational Land Imager and Sentinel-2 Multispectral Instrument collect visible and near-infrared observations at spatial resolutions of approximately 10–30 m. These data support repeated landscape-scale mapping and vegetation-index analysis (Croft et al., 2014; Drusch et al., 2012), although species-level discrimination can remain difficult in heterogeneous vegetation.

Coarse-resolution imagery represents relatively large ground areas within each pixel, commonly hundreds of metres to one kilometre. These products are useful for regional vegetation condition and phenology, but are less suitable for detecting small, sparse or mixed invasive plant patches (Colditz et al., 2011).

Hyperspectral sensors record many narrow spectral bands and can improve discrimination when invasive species have distinctive biochemical, structural or phenological signatures (Clark & Rilee, 2010). Airborne instruments and spaceborne imaging spectroscopy missions offer detailed spectral information, but their use can be limited by data volume, processing requirements, cost and cloud cover (Somers & Asner, 2013b).

Synthetic aperture radar systems, including Sentinel-1, RISAT and RADARSAT, acquire observations under cloudy conditions and variable illumination. Radar measurements are sensitive to surface roughness, vegetation structure and moisture, making them useful complements to optical data in wetland, riparian and structurally complex environments. Species-level interpretation remains challenging because scattering responses are not unique to individual plants.

Light detection and ranging systems mounted on airborne, terrestrial or spaceborne platforms use laser returns to describe three-dimensional vegetation structure, including canopy height and vertical complexity (Coyle et al., 2019; Lefsky & Cohen, 2002). These measurements can complement optical imagery when invasive plants alter canopy or understorey structure.

### RS techniques and analysis tools

#### Vegetation indices

Vegetation indices (VIs) help quantify biomass, chlorophyll content, leaf area index (LAI) and plant condition (Verma et al., 2022). Satellite platforms, including Landsat (NASA, USA), Sentinel-2 (ESA, Europe), MODIS (NASA, USA), together with airborne and UAV-based multispectral and hyperspectral sensors, provide data used to calculate VIs (Anderson & Gaston, 2013). Indices such as NDVI, EVI, soil-adjusted vegetation index (SAVI) and red-edge NDVI provide complementary measures of plant condition, soil background and canopy structure (Jiang et al., 2008). Several IPS exhibit higher greenness or unique phenological timing, including early leaf-out, and prolonged greenness, that can be captured by indices like red-edge based indices or NDVI (Müllerová et al., 2017). Time-series VI data can be used to map invasion spread, monitor seasonal growth patterns and evaluate the ecological impact of IPS over a large landscape (Bradley, 2014). However, spectral confusion in mixed vegetation, index saturation in dense canopies and dependence on atmospheric conditions can limit VI-based IPS detection (Mutanga et al., 2023).

Airborne hyperspectral data provide detailed spectral information for invasive plant mapping. Sabat-Tomala et al. (2022) used HySpex imagery and iterative accuracy assessment to map invasive plants. Underwood et al. (2003) used airborne hyperspectral imagery to map non-native plants in the Sacramento-San Joaquin Delta. These examples show how narrow spectral bands, field reference data and appropriate classifiers can improve species discrimination.

#### Light detection and ranging (LiDAR) processing

Light detection and ranging is an active remote sensing technology that measures the distance between a sensor and the surface to generate three-dimensional representations of terrain and vegetation (Lefsky & Cohen, 2002; Pettorelli et al., 2014). Airborne laser scanning has supported rapid identification of *Acer negundo* in riparian forests (Mielczarek et al., 2023), while combined hyperspectral and light detection and ranging attributes have been used for invasive-shrub mapping in urban environments (Chance et al., 2016). Recent work also demonstrates the value of high-density airborne data for detecting invasive shrub patches (Choi et al., 2025).

#### SAR in invasive species detection

Vegetation and moisture differences between IPS and surrounding vegetation can be detected using SAR data (Rajah et al., 2018). Invasive plants grow very thick or tall stands, or occupy distinctive settings, such as floating mats or flooded zones, creating characteristic backscatter patterns (Bolch et al., 2020). *Phragmites* in wetlands grow as tall, dense reeds with high moisture content; the resulting strong double-bounce and volume scattering yield higher L-Band returns than sparse native marsh, enabling detection (Marcaccio, 2019). Where palms or woody IPS replace mangroves, new vertical structures can alter polarimetric signatures (Shennan & Crabbe, 2024). The sensitivity of synthetic aperture radar to biomass, geometry and moisture can produce a distinctive scattering “fingerprint” for many IPS.

#### Machine learning and deep learning

Machine learning algorithms, particularly supervised classifiers such as random forest, support vector machine and decision trees, are widely used with remote sensing data for invasive plant detection (Anand et al., 2021; Gupta et al., 2024; Zaka & Samat, 2024). Models may combine spectral bands, vegetation indices, texture, phenological metrics and structural variables. Deep learning approaches are also increasingly used with high-resolution and hyperspectral imagery. Table 3 summarises the reported methods and input features; because individual studies may use several techniques, the counts are not mutually exclusive.

**Table 3:** Techniques, sensors, input features and classification approaches reported in the reviewed studies

	Sensor	Vegetation indices^**^(e.g., NDVI, SAVI, etc.)	Per-pixel–based classification (supervised and unsupervised)	Neural network classification	Decision tree classifier	Spectral (raw bands)	References^*^
Aerial photography/UAV	Aerial photographs (2)	-	-	-	-	2	27, 81
	UAV imagery (25)	3	15	-	2	5	1, 2, 4, 6, 7, 15, 16, 17, 31, 32, 42, 44, 48, 59, 70, 71, 74, 79, 89, 94, 95, 97, 102, 107, 144
**Multispectral datasets**							
Medium-resolution imagery	Landsat 5 TM (4)	1	2	1	-	-	25, 87, 91, 164
	Landsat 8 (14)	3	5	2	1	3	5, 26, 58, 62, 68, 77, 105, 113, 116, 121, 129, 142, 148, 165
	MODIS (16)	2	6	-	4	4	32,33, 37, 47, 56, 58, 63, 69, 81, 82, 104, 106, 109, 110, 115, 162
	Sentinel 1,2 (10)	3	5	1	-	1	5, 16, 32, 33, 84, 87, 92, 138, 139, 151
**Hyperspectral datasets**							
	AVIRIS (5)	-	1	-	1	3	35, 58, 118, 135, 159
Spaceborne	Hyperion (6)	1	3	-	1	2	21, 30, 91, 134, 164, 166
**RADAR**							
	Sentinel 2 (5)	-	-	-	-	5	58, 81, 82, 84, 89
	Landsat (8)	-	2	-	-	6	30, 37, 38, 61, 77, 81, 82, 84
	LiDAR (18)	-	5	-	-	13	27, 37, 38, 43, 56, 58, 60, 64, 65, 72, 81, 82, 85, 89, 91, 102, 131

^*^The supplementary data with all the studies used are provided in Appendix A of the supplementary information in the link provided (https://www.scidb.cn/en/anonymous/WkYzZVly)

^**^‘Vegetation indices’ refers to the use of derived spectral indices (e.g., NDVI, SAVI and EVI) as input features in classification or analysis. ‘Spectral’ refers to the direct use of raw spectral band information (e.g., reflectance values from multispectral or hyperspectral sensors) without index derivation

^#^The reported counts are not mutually exclusive; a single study may utilise multiple approaches (e.g., spectral data, vegetation indices and machine learning classifiers such as decision trees or random forest) and is therefore counted across relevant categories

#### Role of species distribution and habitat modelling

Species distribution modelling predicts potential distributions from occurrence data and environmental variables (Franklin, 2013). In invasive plant research, remotely sensed vegetation indices, land cover, topography, climate variables and structural metrics can be incorporated into statistical or machine learning models. Diao and Wang (2014), for example, developed an invasive species distribution model using fine-resolution remote sensing. Such models can support habitat-suitability assessment and identification of areas at risk of future invasion.

#### Time series analysis

Time-series analysis uses repeated observations to identify phenological differences, invasion trajectories and management responses. Multidate Landsat, Moderate Resolution Imaging Spectroradiometer, Sentinel-2 or radar observations can reveal periods when invasive plants differ most clearly from surrounding vegetation (Bradley, 2014). In southern Iran, Izadi, Chamani and Zamani-Ahmadmahmoodi (2022) used a remote sensing time series to examine vegetation responses to the spread of *Prosopis juliflora*.

## Challenges and limitations of remote sensing in IPS detection

Despite significant advancements in remote sensing applications for invasive plant species (IPS) detection, several methodological and practical challenges remain. Spectral confusion between invasive and native vegetation continues to limit classification accuracy, particularly in heterogeneous landscapes where species exhibit similar spectral signatures. Mixed pixel effects further complicate detection, especially when using moderate-resolution satellite imagery, where individual pixels often represent multiple land cover types. Atmospheric conditions, including cloud cover and seasonal variability, can restrict data availability and reduce temporal consistency in monitoring efforts. Additionally, the high cost and limited accessibility of high-resolution hyperspectral and LiDAR datasets pose constraints for large-scale applications. Scalability also remains a challenge, as methods developed for local or regional studies are often difficult to generalise across broader geographic extents due to ecological variability and differences in data availability. These limitations highlight the need for improved data integration, multisensor approaches and robust modelling frameworks to enhance the reliability and applicability of remote sensing–based IPS detection. This review also has limitations. The bibliometric results depend on database coverage, search terms, indexing practices and the inclusion criteria; consequently, relevant studies outside Web of Science and Scopus or studies not using the selected terms may have been missed. Geographic and sensor comparisons are influenced by uneven reporting, and the reported accuracy values were derived from heterogeneous validation designs, so direct comparisons should be interpreted cautiously. Manual coding of impacts and Sustainable Development Goal linkages also involves judgement, despite the use of predefined categories.

## Conclusion

This review synthesises 25 years of research (2000–2025) on remote sensing for detecting, monitoring and managing invasive plant species. The evidence shows substantial progress through higher-resolution multispectral and hyperspectral imagery, uncrewed aerial systems, light detection and ranging, synthetic aperture radar, predictive modelling and machine learning. Bibliometric and temporal analyses also show a shift from early concentration in North America and Europe toward increasing contributions from Asia, Africa and other previously underrepresented regions.

However, important limitations remain despite these advances. Spectral confusion between invasive and native species, limited availability of labelled datasets and uneven geographic distribution of research still limit model performance and applicability. Moreover, the effectiveness of RS-based methods is still limited by technical challenges, such as cloud cover, trade-offs in resolution and difficulties in detecting sparse or understory invasions. These limitations emphasise the need for integration of RS with field-based observations, ecological expertise and complementary analytical methods.

Current and emerging satellite missions including EnMAP, PRISMA and NISAR together with advances in small satellite constellations and multimodal data integration are expected to improve spectral, spatial and temporal monitoring capabilities. Furthermore, the integration of citizen science and crowdsourced data provides new avenues for enhancing validation and increasing data availability. Current barriers may be reduced through improved access to data, affordable processing frameworks and increased interdisciplinary collaboration between the fields of remote sensing, ecology and environmental policy. The understanding of the spatiotemporal dynamics of invasive plant species is ultimately a critical component of the development of targeted, region-specific management strategies and proactive policy interventions. Improving these capacities will be important for mitigating ecological impacts and supporting biodiversity conservation in sensitive and data-sparse areas.

## Supplementary Information

Below is the link to the electronic supplementary material.

ESM 1 (DOCX 4.11 MB)

## Data Availability

The authors confirm that the data supporting the findings of this study are available within the article and its Supplementary material (https:/www.scidb.cn/en/anonymous/WkYzZVly). Raw data that support the findings of this study are available from the first author, upon request.

## Appendix A.

Additional material to this article can be found freely accessible online at https://www.scidb.cn/en/anonymous/WkYzZVly.
