# Supplementary material for: Technological approach to invasive species: the role of remote sensing and predictive modelling
Source: Environ Monit Assess. 2026 Oct 31;198(11):1270. doi: 10.1007/s10661-026-16070-x (PMC13723693; doi:10.1007/s10661-026-16070-x)
Supplement: Supplementary file 1 [file 10661_2026_16070_MOESM1_ESM.docx]

Table S1. Regional distribution of studies using Landsat and Sentinel-2 multispectral imagery

| **Region** | **Share of sensor-specific studies (%)** | |
| --- | --- | --- |
|  | **Landsat** | **Sentinel-2** |
| North America | 32 | 25 |
| China | 20 | 6.25 |
| Africa | 12 | 31.25 |
| Global | 12 | 6.25 |
| India | 12 | 6.25 |
| Asia | 4 | - |
| Australia | 4 | - |
| Europe | 4 | 18.75 |
| South America | - | 6.25 |

Note: Percentages were calculated within each sensor-specific subset (Landsat, n = 25; Sentinel-2, n = 16). India and China are reported separately from the broader Asia category, consistent with the manuscript.

Table S2. Frequency of the most commonly reported invasive plant species in the 41-study subset

| **Species** | **Percentage of Studies (%)** |
| --- | --- |
| *Eichhornia crassipes* | 19.51 |
| *Prosopis juliflora* | 17.07 |
| *Acacia longifolia* | 9.76 |
| *Spartina alterniflora* | 9.76 |
| *Acacia dealbata* | 7.32 |
| *Chromolaena odorata* | 7.32 |
| *Lantana camara* | 7.32 |
| *Parthenium hysterophorus* | 7.32 |
| *Tamarix spp.* | 7.32 |

Note: Percentages were calculated using the 41-study species-frequency subset. Only the most frequently reported species are listed, so percentages do not sum to 100%.

Table S3. Regional distribution (%) of studies on invasive plant species using remote sensing

| **Region** | **Percentage of Studies (%)** |
| --- | --- |
| India | 21.95 |
| North America | 9.76 |
| Africa | 14.63 |
| Europe | 14.63 |
| Asia | 7.32 |
| Australia | 7.32 |
| Global | 7.32 |
| China | 2.44 |
| South America | 2.44 |
| Tropical | 2.44 |

* Note: Percentages were calculated using the 41-study supplementary subset. The listed categories account for 37 records; four subset records are not represented in these regional categories. India and China are reported separately from Asia.
